# Environmental asbestos exposure and clustering of malignant mesothelioma in community: a spatial analysis in a population-based case–control study

**DOI:** 10.1186/s12940-021-00790-3

**Published:** 2021-09-15

**Authors:** C. Airoldi, C. Magnani, F. Lazzarato, D. Mirabelli, S. Tunesi, D. Ferrante

**Affiliations:** 1 Medical Statistics, Department of Translational Medicine, Università Degli Studi del Piemonte Orientale, Via Solaroli 17, 28100 Novara, NO Italy; 2 Cancer Epidemiology Unit, CPO-Piemonte, Novara, Italy; 3 Interdepartmental Center for Studies on Asbestos and Other Toxic Particulates “G. Scansetti”, University of Turin, Turin, Italy; 4 Unit of Cancer Epidemiology, “Città Della Salute E Della Scienza” Hospital, Turin, Italy

**Keywords:** Asbestos, Mesothelioma, Spatial analysis

## Abstract

**Background:**

Neighborhood exposure to asbestos increases the risk of developing malignant mesothelioma (MM) in residents who live near asbestos mines and asbestos product plants. The area of Casale Monferrato (Northwest Italy) was impacted by several sources of asbestos environmental pollution, due to the presence of the largest Italian asbestos cement (AC) plant. In the present study, we examined the spatial variation of MM risk in an area with high levels of asbestos pollution and secondly, and we explored the pattern of clustering.

**Methods:**

A population-based case–control study conducted between 2001 and 2006 included 200 cases and 348 controls. Demographic and occupational data along with residential information were recorded. Bivariate Kernel density estimation was used to map spatial variation in disease risk while an adjusted logistic model was applied to estimate the impact of residential distance from the AC plant. Kulldorf test and Cuzick Edward test were then performed.

**Results:**

One hundred ninety-six cases and 322 controls were included in the analyses. The contour plot of the cases to controls ratio showed a well-defined peak of MM incidence near the AC factory, and the risk decreased monotonically in all directions when large bandwidths were used. However, considering narrower smoothing parameters, several peaks of increased risk were reported. A constant trend of decreasing OR with increasing distance was observed, with estimates of 10.9 (95% CI 5.32–22.38) and 10.48 (95%CI 4.54–24.2) for 0–5 km and 5–10 km, respectively (reference > 15 km). Finally, a significant (*p* < 0.0001) excess of cases near the pollution source was identified and cases are spatially clustered relative to the controls until 13 nearest neighbors.

**Conclusions:**

In this study, we found an increasing pattern of mesothelioma risk in the area around a big AC factory and we detected secondary clusters of cases due to local exposure points, possibly associated to the use of asbestos materials.

**Supplementary Information:**

The online version contains supplementary material available at 10.1186/s12940-021-00790-3.

## Background

Asbestos is the only established causal factor for pleural and peritoneal malignant mesotheliomas (MM) and one of the main occupational risk factors for lung cancer. The risk of cancer increases with intensity, duration, and frequency of exposure to asbestos [1].

A large body of literature exists on the risk of MM after occupational exposures to asbestos, as summarized in several reviews [1–3]. The consequences of para-occupational exposures, i.e. those experienced by family members of asbestos workers, have also been widely studied [4, 5]. Finally, neighborhood exposures had already been associated with MM by Wagner et al. [6]; later, some epidemiological studies detected the increased MM risk in residents living near asbestos mines and asbestos products factories [7–17].

Italy, due to long-term asbestos use and consequent excess of MM incidence and mortality, is one of the countries more sensitive to the issues of monitoring and preventing asbestos-related health effects. Research focused on the area of Casale Monferrato (northwest Italy), where the largest Italian asbestos cement (AC) plant, owned by Eternit, was active from 1907 to 1986. The size of the work force varied over time and was up to 1500 workers. In 1981 the company reported the use of 15,000 tons of asbestos (10% crocidolite) [18]. In this area an extremely high incidence of MM was observed (more than 10 times higher than the corresponding Italian incidence rates), [19] and several studies have been conducted regarding the AC workers [20], their wives [21] and the residents [22]. Moreover, Magnani et al. [23] and Maule et al. [14] in a case control study including cases diagnosed in 1987–1993 provided strong evidence of an increased MM risk related to the distance of dwellings from the Eternit plant, suggesting a causal role of the asbestos environmental pollution. Residents near the plant had an odd ratio (OR) for mesothelioma of 10.5 (95% CI 3.8–50.1), adjusted for occupational and domestic exposure. Furthermore, the results of spatial clustering tests gave some support for the hypothesis of exposure (and risk) associated with secondary sources of asbestos.

Such secondary sources included some separate, accessory departments of the factory (i.e. the finished products warehouse and rail yard, both located on the eastern side of downtown Casale Monferrato while the factory was on its western border), the transport systems across the town of raw asbestos and AC products and a landfill for asbestos wastes discharge. They also included private buildings, yards and lanes where AC tailings from the work process had been incorrectly disposed of: for instance, to cover small—and sometimes large—land areas after having been finely broken, or as fine dust (locally known as “polverino”) as attic insulation [24].

We wished therefore to investigate clustering consequent to this exposure, by taking advantage of the more recent and larger dataset of our 2001–2006 case–control study [22]. We also wanted to overcome some limitations of the analyses of our first case–control study, namely the fact that the estimates of environmental exposures had been adjusted for occupational and domestic exposure by classifying such exposures only in a dichotomous way and that allowance had been made only for a fixed lag time period of 20 years.

This study analyzed individual data generated by a case–control sampling design aiming to i) assess the spatial variation of MM risk in the asbestos exposed area of Casale Monferrato and ii) detect clustering associated to the secondary sources of asbestos.

## Methods

### Study design

The present analyses used the data collected in a case–control study on pleural MM conducted in Casale Monferrato Local Health Authority (LHA) between 1 January 2001 and 30 June 2006 [22]. The LHA is the basic administrative unit of the Italian National Health Service. At the time of the study, Casale Monferrato LHA included the town of Casale Monferrato and the surrounding area, for a total of 60 towns and villages of different size; the total resident population on December 31, 2001 was 117,680, (of whom 35 238 lived in Casale Monferrato). Population rosters of residents, maintained by the LHA for administrative purposes, were used to assess eligibility of cases and to sample controls.

Cases of histologically verified MM of the pleura were actively searched in the departments of pathology, respiratory medicine, oncology, internal medicine, thoracic surgery and radiotherapy of the hospitals serving the study area. Controls were randomly selected from the population rosters of the LHA of Casale Monferrato. Two hundred twenty-three incident cases and 552 age (± 18 months) and gender- individually matched controls were identified. To increase power in the younger age classes, the case control ratio was 1:2 for cases 60 years and older, and 1:4 for younger cases. Each subject (or the next-of-kin for decedents or subjects unable to answer) was interviewed on demographic characteristics, lifelong occupational and residential history and other information related to asbestos exposure, including that of relatives and cohabitants. A structured questionnaire was used. Likelihood and intensity of asbestos exposure were estimated from experts, based on the interview information. As individuals usually had multiple exposure circumstance (work and not-work related), the assessment took into account their whole exposure history and computed cumulative exposure indexes, reflecting the contribution of all sources. Cumulative exposure was expressed in fibres/mL-year. More details on data collection procedures and exposure assessment have been published elsewhere [22].

### Geographical classification

All residential addresses reported at interview, generally more than one for each subject, were geocoded as Universal Transverse Mercator (UTM) geographic coordinates using a Global Positioning System (GPS) accessed from Google Maps. Addresses missed at first instance were manually checked, amended whenever appropriate, and resubmitted to geocoding process. The geographic coordinates of the AC factory location were determined in a similar manner. Moreover, geographical coordinates of Casale Monferrato and towns and villages of LHA’s boundaries were downloaded from the Italian National Institute of Statistics [25]. The factory is in the NW area of Casale Monferrato town, at about 1500 m from the center and 250 m from the closest residential areas.

The dwelling address was chosen as a proxy to residential exposure. The dwelling with the longest residency duration (main residence) after excluding those outside the LHA boundaries was used as the location of study subjects for the analysis. The time window of interest was defined excluding the last 20 years before diagnosis to exclude any exposure occurring during the preclinical phase of the disease. The 20 years period was chosen as an extreme value, following the previous analyses in the same setting [14, 23]. As the duration of that interval is not known with precision for MM we also conducted analyses with consideration of shorter periods, in particular using the 10 years period customarily used in other studies [16, 26]. A further sensitivity analysis was carried out using as exposure proxy the closest residence, with reference to the distance from the Eternit factory.

### Statistical analysis

Categorical variables were summarized using absolute and percentage frequencies, while mean and standard deviation or median and interquartile range were used for numeric variables. Results were presented for all subjects and separately for cases and controls.

The geodetic distances of each subject location to the AC industry (pollution source—foci) were calculated. Maps with point locations, AC plant and Casale Monferrato boundaries were produced. Analyses were focused on the Casale Monferrato LHA, and residential addresses outside it were disregarded.

Unconditional multivariable logistic regression models, as suggested by Pearce [27], were used to assess the relationship between distance from AC plant and the probability of being a case, adjusted for gender, age at diagnosis and type of interview, following Ferrante et al. [22]. Distance was considered in continuous and categorical way, choosing different thresholds (500 m, 3 km, 5 km). Moreover, to adjust for occupational and domestic asbestos exposure, the individual estimate of cumulative exposure previously calculated by experts using interview information were included in the models as either continuous covariates (in fibre/mL-year) or dichotomous categories. Odds ratios (OR) and 95% confidence intervals (95% CI) were reported.

To explore the spatial distribution of cases’ and controls’ locations, in search of spatial aggregations, first- and second- order properties were assessed. The former investigates intensity and spatial density measuring the distribution of events in the study region. The latter gives information on the interaction between cases and controls in terms of clustering ability. Finally, scans of local case/control ratios and nearest-neighbor statistics were calculated.

To detect local differences between the spatial pattern of cases and of controls (first-order property), different approaches were considered. Bivariate Gaussian Kernel density estimations were used considering different smoothing parameters (bandwidths). Global test of clustering based on Kelsall and Diggle method [28] was also performed. Finally, point source pollution models (raised incidence models) were implemented to allow for a more flexible parametric exposure modelling [29]. These models describe natural spatial variation in intensity and possible raised risk around a pre-specified point as the putative source of environmental pollution (i.e.: the AC plant).

Secondly, we evaluated the general tendency for cases to occur more closely together than expected from random sampling (second-order property). The D function [30] was calculated as the difference between the functions that measure the number of cases/controls up to a given distance from any particular case/control, for cases and controls respectively. Significant difference from 0 means that there is a difference in the distribution of cases and controls, with clustering occurring at the range of those distances. The D function was drawn with its pointwise envelopes to explore at what distances any observed clustering tend to occur, average over the entire study area [31].

To determine any local areas where the observed ratio of cases/controls (number of cases compared to the number of controls) appeared inconsistent with the ratio observed in the rest of the study area, the Kulldorf test was used. Particularly, this scanning local rate approach was performed to find the most unusual aggregations of cases adjusted for multiple testing considering circular windows with variable radii [32]. Lastly, the Cuzick Edward test (nearest-neighbor statistic) was assessed to examine local patterns of cases in the vicinity of other cases [33]. Evidence for clustering was evaluated for 3, 5, 7, 9, 11, 13 and 15 nearest neighbors, chosen ‘a priori’.

Sensitivity analyses excluding the time window of 10 years before diagnosis and using the dwelling at the nearest distance from the AC plant were performed to assess for different lag time periods and different selection of exposed dwellings. Unconditional logistic models were further adjusted for the individual estimate of cumulative asbestos due to environmental exposures, in addition to that stemming from occupational and domestic assessment, already included in our main analyses. The estimated environmental exposure level only partially overlapped with exposure index based on distance from the Eternit factory, as the quantitative assessment protocol took into account all sources of exposure. Moreover, only residential distances up to 2 km from the Eternit factory, 1 km from the warehouse/rail stock or 100 m from the transport path, whichever the closest, were considered. More details on the how the exposure was calculated are available elsewhere [22]. Statistical significance was set at 0.05; 999 simulations were performed when Monte Carlo approach was used. SAS 9.4 was used to manage the database, while the models implementation, the spatial analysis and the tests were performed with STATA and R (packages spatstat, mgcv, MapGAM, splancs).

## Results

**Fig. 1:**
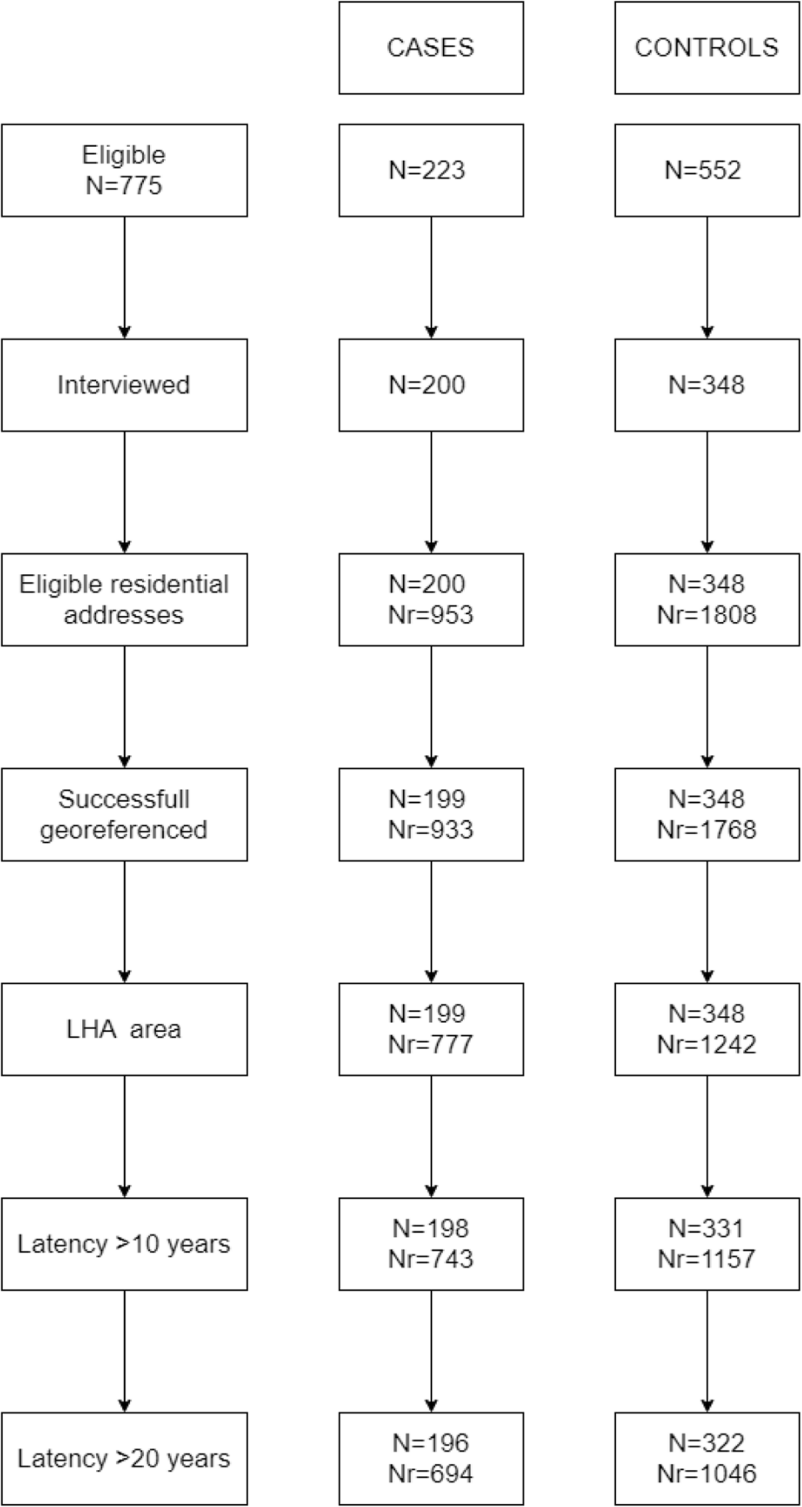
Case control study on MM in Casale Monferrato area. Flow chart of subjects included in the analysis. N indicates the number of subjects, Nr the number of dwellings. LHA = Local Health Authority

The study base included 223 eligible cases (mean age: 68.4; SD: 11.3; males: 62%) and 552 controls (mean age: 65.4; SD: 12.1; males: 61%). Two hundred cases (89.7%) and 348 (63%) controls accepted the invitation and were interviewed (Fig. 1). Distribution by sex of participating cases and controls was similar, while cases were older than controls because of the oversampling of controls matched to cases under 60 and the different age distribution of non-participating controls [22]. A higher level of education was associated with response [34].

Two thousand seven hundred sixty-one dwellings were reported at interview and were automatically geocoded (953 for cases and 1808 for controls; average number of dwellings per person: 4.8 for cases and 5.2 for controls); for about 5% (127/2761) of them a manual check was necessary to amend errors and imprecisions that precluded automatic georeferentiation. After excluding the few dwellings for which full address was not available (20/953 = 2.1% for cases and 40/1808 = 2.2% for controls) and those outside the LHA area (156/933 = 16.7% for cases and 526/1768 = 29.8% for controls), geographic coordinates were available for 777 and 1242 addresses, corresponding to 199 cases and 348 controls. The application of the 10 and 20-years lags reduced the number of subjects eligible for the analyses to 198 cases and 331 controls, and to 196 cases and 322 controls, respectively (Fig. 1).

**Fig. 2:**
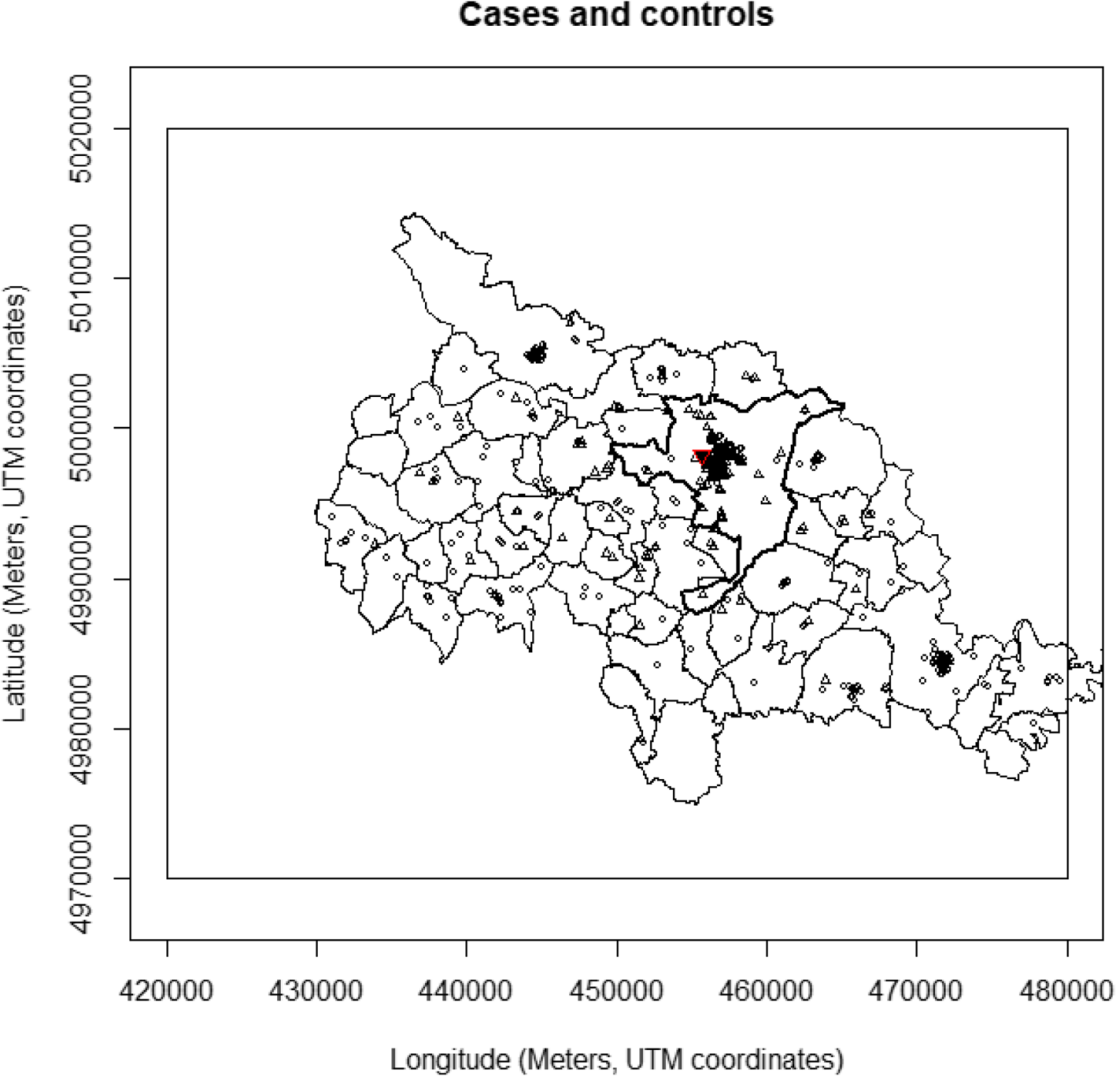
Case control study on MM in Casale Monferrato area. Spatial distribution of the residences of cases (triangles) and controls (circles) in the geographic area of LHA (solid line), approximately 2500 km^2^ around Casale Monferrato (bold solid line). Longest-held residences after excluding 20 years before the date of diagnosis of the index case. The location of the AC plant is also indicated (red triangle). [R Spatstat]

Figure 2 presents the distribution of index residences and the borders of the municipalities forming the Casale Monferrato LHA. The spatial distribution of the controls on the map (circles) represents the population density. The square covers a 2500 km^2^ surface (50 × 50 km).

Table 1 presents results on MM risk by classes of residential distance, both considering all subjects and excluding subjects occupationally exposed. For ‘all subjects’ category, the median distance from residence to AC factory was 2.10 [IQR 5.92] and 11.32 [IQR 15.77] km for cases and controls, respectively. Cases lived more frequently near the AC plant and 64.3% of them had the longest-held residence within 5 km from the plant, compared to 34.5% of controls. Considering only non-occupationally exposed subjects (Table 1 bottom section), closely similar results were obtained: distances from the AC factory were lower in cases than controls (2.03 [IQR 4.59] vs 10.36 [IQR 15.86]), 71.95% of cases and 38.12% of controls had the index dwelling within 5 km from the factory. The analyses considering all subjects showed a constant trend of decreasing OR with increasing distance from the factory, both using distance as categorical or numerical variable. In distance classes up to 10 km from the factory (reference > 15 km) a tenfold increase of risk was observed, statistically significant. The model adjusted for age, sex and type of interview showed similar ORs compared to that adjusted also for occupational and domestic exposure but AIC index was smaller. OR for 1 km increasing distance were 0.87 (95% CI 0.84–0.91) and 0.88 (95% CI 0.84–0.91), respectively. Similar results were observed when the analyses were restricted excluding subjects with occupational exposure (lower part of Table 1), confirming that the risk relation with distance to the factory was not a spurious result and cannot be explained by the effect of occupational exposure.

**Table 1:** Case control study on MM in Casale Monferrato area. Risk of MM of the pleura in relation to the distance of longest-held residence (after exclusion of 20 years before the date of diagnosis) from the AC plant. Absolute and relative frequencies of distance categories and median [interquartile range] of distance. Logistic models adjusted by age, sex, type of interview (*) and age, sex, type of interview and occupational and domestic asbestos exposure as continuous covariate (**) or age, sex, type of interview and domestic asbestos exposure as continuous covariate (***); odds ratios (OR), 95% confidence intervals (in brackets) and Akaike Information Criterion (AIC)

All subjects					
**Distance from the AC plant (km)**	**All** ***N* = 518**	**Cases** ***N* = 196**	**Controls** ***N* = 322**	OR adjusted*	OR adjusted**
0–5	237 (45.75)	126 (64.29)	111 (34.47)	10.91 (5.32–22.38)	10.81 (5.26–22.21)
5–10	75 (14.48)	40 (20.41)	35 (10.87)	10.48 (4.54–24.2)	10.42 (4.51–24.07)
10–15	85 (16.41)	18 (9.18)	67 (20.81)	2.2 (0.9–5.33)	2.2 (0.9–5.34)
> 15	121 (23.36)	12 (6.12)	109 (33.85)	Ref	Ref
**AIC**				513.71	515.43
Distance, Km	6.06 [12.25]	2.10 [5.9]	11.32 [15.77]	0.87 (0.84–0.91)	0.88 (0.84–0.91)
**AIC**				514.91	516.61
Non-occupationally exposed subjects					
**Distance from the AC plant (km)**	**All** ***N* = 263**	**Cases** ***N* = 82**	**Controls** ***N* = 181**	OR adjusted*	OR adjusted***
0–5	128 (48.67)	59 (71.95)	69 (38.12)	13.73 (4.57–41.32)	12.66 (4.19–38.28)
5–10	30 (11.41)	12 (14.63)	18 (9.94)	8.5 (2.29–31.61)	7.15 (1.87–27.36)
10–15	39 (14.83)	6 (7.32)	33 (18.23)	2.81 (0.7–11.29)	2.47 (0.61–10.04)
> 15	66 (25.10)	5 (6.10)	61 (33.70)	Ref	Ref
**AIC**				251.83	249.96
Distance, Km	5.29 [13.56]	2.03 [4.59]	10.36 [15.86]	0.86 (0.81–0.91)	0.86 (0.81–0.91)
**AIC**				247.18	244.89

Results using different distance categories (0–3, 3–5, 5–7, 7–9, 9–11, 11–13, 13–15, > 15 km and 0–500, 500–1000, 1000–1500, 1500–2000, > 2000 m) were reported in Supplementary Table S1.1 and are consistent with the main analyses, although the trend was less regular and CIs were wider, as expected because of the smaller numbers of subjects in these smaller categories. No controls lived in a radius of 500 m while 8 cases did, including 3 without occupational exposure. Subjects with residences in the 500–1000 m band showed an increased risk: OR was about 14 (all subjects analyses) or 18 (exclusion of subjects with occupational exposure) (Supplementary Table S1.2). In order to further control for the possible role of occupational, domestic and total (sum of occupational, domestic and environmental) asbestos exposure, specific analyses were performed by including exposure estimates in the model, using different metrics (Table S1.3). Results remained very close to those from the main analysis.

**Fig. 3:**
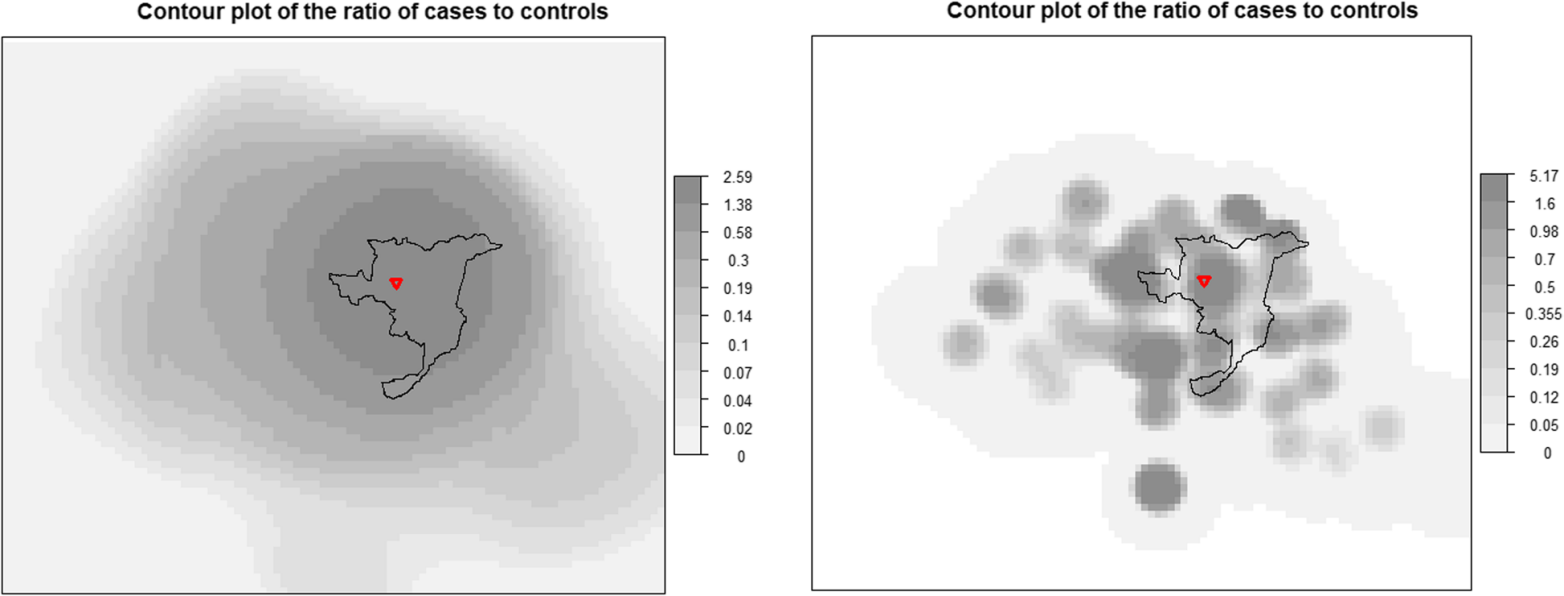
Case control study on MM in Casale Monferrato area. Contour plot of the kernel density surfaces of the case to control ratio, including occupationally and non-occupationally exposed individuals in an area of approximately 2500 km2 around Casale Monferrato (solid line). Bivariate Gaussian Kernels with smoothing parameters set to 10 and 20 km for cases and controls, respectively, in the left panel and 2 and 4 km in the right panel. The location of the AC plant (red triangle) is indicated. The legend reports the value of ratio and the corresponding grey shades

**Fig. 4:**
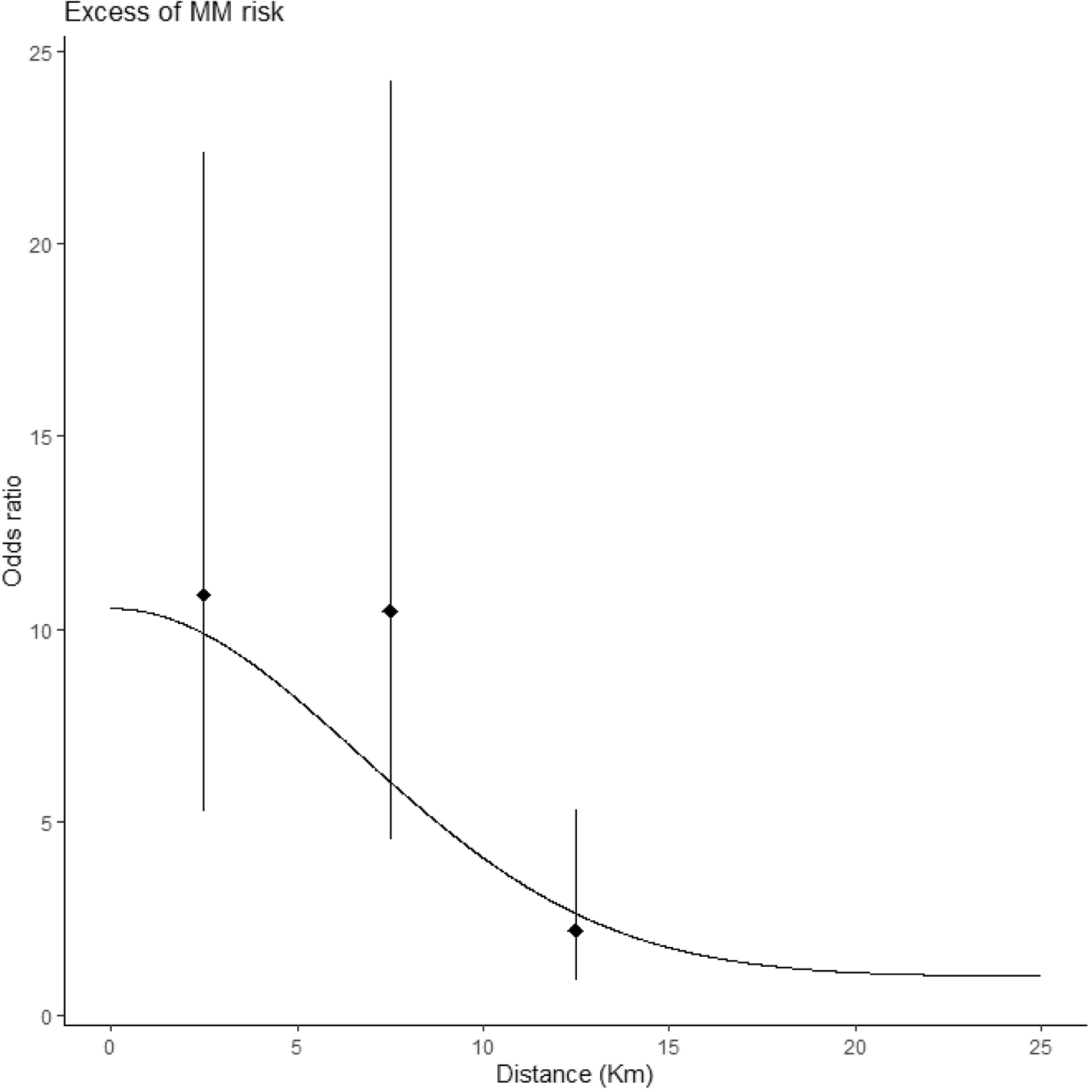
Case control study on MM in Casale Monferrato area. Risk of MM of the pleura in Casale Monferrato in relation to the distance of individuals’ longest-held residence (after exclusion of 20 years before the date of diagnosis) from the AC plant. Risk estimates are adjusted for age, sex, type of interview. Odds ratios and 95% confidence intervals estimated through the logistic model are represented by error bars while those estimated through the model with exponential decay of the risk by distance are shown as a smooth line

As observed in Fig. 2, events were not randomly allocated: the majority of subjects lived in the center of Casale Monferrato or in the surrounding urban areas. The contour plot of the ratio of Kernel density functions for cases and controls based on the Kelsall and Diggle method [35] was obtained (Fig. 3). We observed (left panel) a well-defined peak near the AC factory, and risk seems to decrease monotonically in all directions when bandwidths were set at 10 and 20 km for cases and. The area around the Eternit was associated with a significantly increased risk, and the global test yielded a p-value of 0.001, indicating that the overall difference between intensities supports the idea of a global pattern of clustering. However, when we reduced the bandwidth values (2 and 4 km for cases and controls, respectively), large effect on the smoothness was observed resulting in a bumpy surface (Fig. 3, right panel). This result must be taken with caution as statistical significance was not reached (*p* = 0.656) and most local clusters were based on few subjects with little ‘a priori’ evidence of exposure hotpoints. Finally, raised incidence models were implemented to investigate the possible elevation in disease risk around the putative source. Estimates of the point source models without covariates were alpha = 14.49 and beta = 0.012, where alpha represents residential excess risk at the source and beta represents risk decay rate per unit squared distances (km) moving away from the source. Considering the adjusted models, the alpha and beta parameters were respectively 9.54 and 0.011 (age, sex, type of interview) and 0.90 and 0.002 (age, sex, type of interview, occupational and domestic asbestos exposure). In terms of deviance (or log-likelihood), the best model is the second (adjusted for age, sex, type of interview). When the analysis was restricted to non-occupationally exposed cases and controls, the model without covariates had an excess risk at the source of 11.64 and the risk decay rate was 0.013 for km. The model with covariates did not converge for collinearity. Comparison between ORs estimated using categorical distance from the source and the raised incidence model, is shown in Fig. 4.

**Fig. 5:**
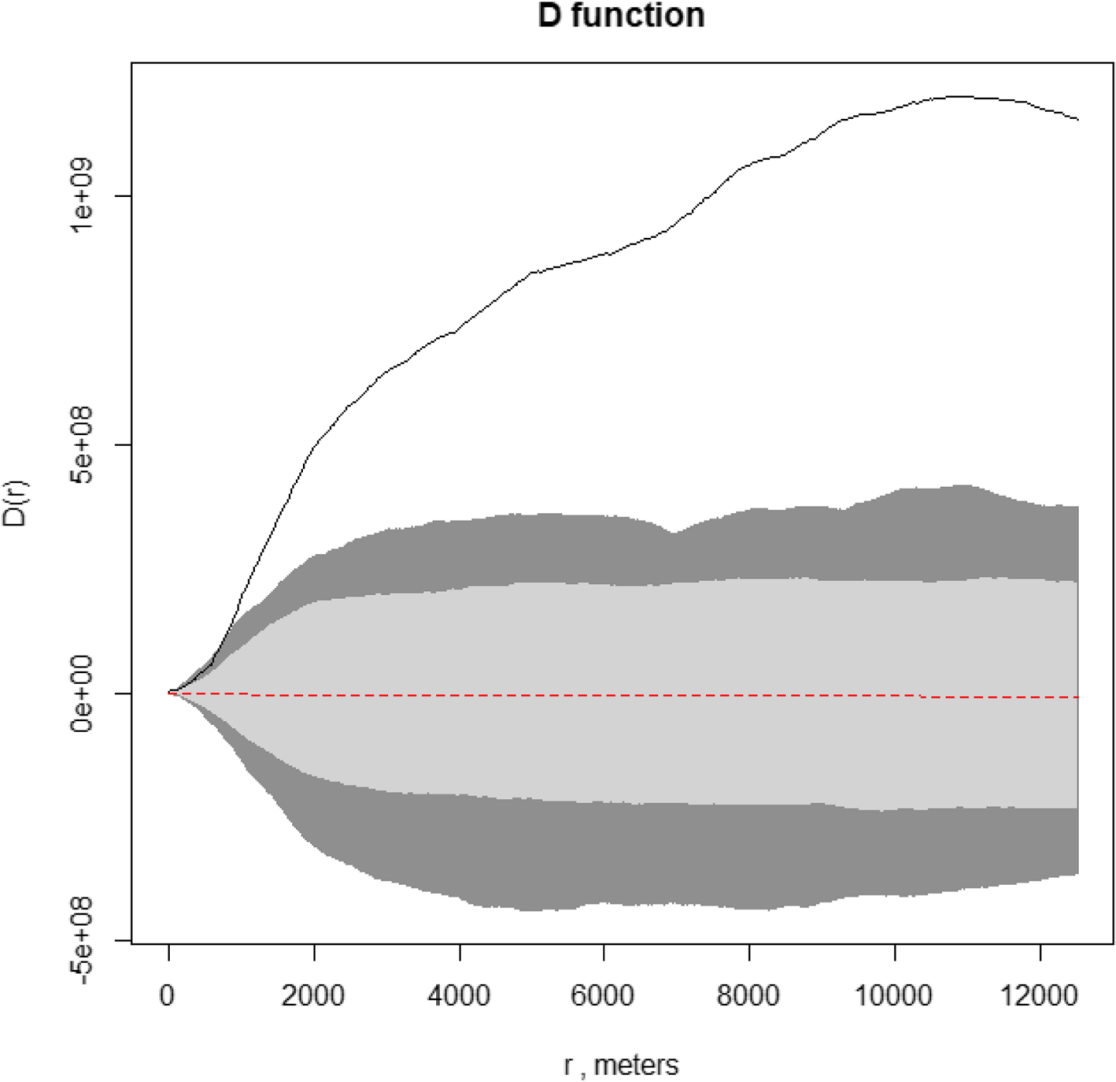
Case control study on MM in Casale Monferrato area. D function. The solid black line represents the observed value, while the red dashed the expected. The grey area reports the envelopes (n simulation = 999) and the lighter grey part the 95% envelopes

The D function, that compared the distributions of cases and controls, provided evidence that their spatial distributions were different as the empirical D function was above the upper limit of the tolerance envelope (Fig. 5). In details, significant evidence of clustering occurred approximately after 700 m, so for any distance greater than this threshold, cases tend to be more aggregated than controls. This conclusion is heavy consistent with the Diggle test results (*p* = 0.001) as it supports the hypothesis that spatially close groups of cases occur more frequently than is consistent with completely random occurrence of the disease among members of the population at risk.

The Kulldorf test identified an area with a significant excess of cases (*p*-value = 0.001) centered at 1.71 km South from the Eternit plant, with a radius of 8.64 km that included 168 cases and 146 controls (Supplementary material, figure S1). The cluster covered almost the whole city of Casale Monferrato and this area was very similar to the area with increased risk obtained using the Kelsall and Diggle method [35] as reported in Fig. 3 (left panel).

Finally, we applied the Cuzick and Edward statistics that calculated for each q nearest-neighbor the number of those that are cases. Table of test statistics and associate p-values both with contrasts for a variety of values of q is presented in supplementary materials (Table S2). Since all the contrasts, except the last (15 vs 13), suggested significant clustering, we conclude that the significant clustering observed among collections of more than thirteen nearest neighbors is driven primarily by clustering among the first thirteen nearest neighbors.

All the analyses were repeated considering a 10 years’ lag period, including 198 cases and 331 controls. The results of the analyses using distance, either categorical or continuous, were reported in table S3.1 (supplementary material) and no relevant differences were observed compared to the main analyses. Also the cluster analyses using Kulldorf, and Cuzick and Edward tests provided unchanged results.

The analyses using the dwellings at the shortest distance from the factory are presented in Supplementary Table S3.2 and again no relevant differences from the main analyses were observed, although a smoother decline of ORs with distance was noticed.

## Discussion

In this study we found a pattern of increased MM risk in the area around a large AC factory and we detected secondary clusters of cases, of smaller size and possibly associated with the local improper reuse of asbestos waste materials. Overall, Kernel estimates with wide bandwidths (10–20 km) and Kulldorf test, were able to identify a large area with increased risk in the city of Casale Monferrato, around the AC plant. However, when narrow bandwidths (2–4 km) were used and the Cuzick Edward test was performed, we observed a different pattern of cases and controls distributions: several peaks of MM risk were spread over the area, not only around the AC plant, and cases tend to aggregate in smaller groups.

The first aim of this study was to explore the spatial variation of MM risk in the area of Casale Monferrato. Using different graphical and statistical methods, we found an increased risk of MM near the AC plant; models that included the distance as covariate showed that risk decreased as distance increased, with a significant excess of cases up to 10 km from the AC plant. A similar inverse relation between distance from a recognized asbestos pollution source and MM risk was observed in two other Italian spatial case–control studies, respectively in the same area of Casale Monferrato [14, 23] and Bari [15]). In our previous study in Casale Monferrato, that included 103 cases and 272 controls, bands of approximately 2 km had been used and significant ORs within 11 km had been estimated for occupationally and non-occupationally exposed subjects [14, 23]. A mixed additive-multiplicative model had been used estimating a RR of 10.5 for residence at zero distance from the source, and a risk decay rate of 0.11 × 10^–7^ per unit squared-distances (meters) moving away from the source. Results had been, thus, very similar to the present study, despite some differences in statistical modelling: in the previous study [14] we had adjusted for occupation in the AC industry and indoor or outdoor domestic exposure to asbestos, while in the current analyses we choose, based on deviance indicator, the model without asbestos exposure covariates. The Bari study involved the geographical area where the Fibronit AC factory had been active for about 50 years. It included 48 cases and 273 controls without occupational, domestic, and household asbestos exposure. A significant OR of 5.29 (95%CI 1.18–23.74) was observed for residential distance < 500 m from the AC plant (reference: distance > 2000 m), while for intermediate distance bands the confidence intervals were very wide and included unity. Our results present higher ORs: in our analysis no controls and 8 cases lived in the first band (infinite risk) and subjects within 500–1000 m (14 cases and 4 controls) had a high and significant OR. These differences might be partly explained by the different selection of the reference category (> 2000 m in Bari, > 15 km in the present study). However, the two areas are different: for the Fibronit AC factory in Bari the distance from the closest residential areas was less than 50 m, compared with about 200 m in Casale Monferrato, but the Fibronit AC factory was much smaller than the Eternit plant, employing a maximum of 400 workers against about 1500. Our contour plot is in line with the map proposed by Maule et al. [14] when the smoothing parameters are the same.

After decreasing the bandwidth values different findings were observed, as the results are strongly dependent on the parameters selection. Using narrow bandwidths, we were able to decompose the global peak observed in the city of Casale Monferrato in four areas with an increased risk of 4 or more. This observation is consistent with the ‘a priory’ information on the occurrence of local point sources of exposure, either associated to the main AC plant or to the use of production residues.

Other studies supported the view than environmental asbestos exposures entail an increased MM risk, despite using a different methodology—as generally they were population surveys where the standardized incidence or mortality ratio (SIR and SMR respectively) was calculated. In Broni, another Italian town where a large AC factory operated, an overall excess of MM cases above the regional average was observed for non-occupationally exposed people and MM incidence was similar to that observed in Casale Monferrato [36]. In Amagasaki (Japan) Kurumatani and Kumagai [11] observed that among persons who had lived within a 300 m radius of an asbestos products factory, the MM SMR was 13.9 (95% CI 5.6–28.7) for men and 41.1 (95% CI 15.2–90.1) for women; the town districts with a significantly elevated SMR reached 2,200 m from the plant.

It is worth noting that the Third report from the (Italian) National Mesothelioma Registry (ReNaM) included an historical outline of the Italian asbestos-cement industry, describing 37 plants, whereas reports on the occurrence of MM after residential exposures have been published only for those of Casale Monferrato, Bari and Broni. These factories had some distinctive features: all used crocidolite in all formulations, produced – among the other articles – pressure pipes, whose formulation required larger amounts of crocidolite, were old (the three oldest in Italy), large (two of the three largest), close to urban areas with a large resident population and are known to have dumped asbestos-cement waste materials at uncontrolled sites accessible to the population and even distributed them for free, to be used by residents for paving roads, yards and, in general, private estates. We consider them, therefore, the most likely ones to have large impact on residents, but also the best suited to be the object of formal epidemiological studies. The lack of comparable studies for the other plants cannot be considered as evidence of no effect on nearby people.

The second purpose of our study was to detect clustering and to find unusual aggregations of cases. Overall, we were able to demonstrate that cases tend to aggregate more than controls, and to observe a significant local cluster of 168 cases, in the area surrounding the AC plant, and consistent with analyses by distance bands. Coherent with the other Italian studies, significant p-values were obtained testing the alternative hypothesis of clustering [14, 15]. Despite the fact that the Kulldorf test was not sensitive enough to detect other clusters, we posit that minor aggregations of cases are incorporated into this major cluster, as already supposed by Maule et al. [14]. Indeed, the results of the Cuzick Edward test indicated than cases tend to significant aggregate up to 13 elements, which suggests the existence of multiple environmental point sources of exposure. It is realistic to suppose that the nature of such large aggregations is domestic and environmental; it is likely to result either from the diffusion of asbestos fibres when the factory was in operation or to be caused by proximity to its transport pathways, as it was centered on the Casale Monferrato main residential area and its extension was such to include the factory, rail-yard, warehouse and dumping site located West, East and North, respectively, of the central residential area. The use of asbestos tailings in buildings, paving of squares, courtyards and roads and other similar instances is also likely to have contributed, as access to waste materials was arguably easier for the closest residents [8, 9, 37, 38]. We may offer a tentative explanation for two further clusters. The first is East of Casale Monferrato and appears to be centered on the residential area of Frassineto Po, a small town where from the nineteen-fifties to the nineteen-seventies a family-based enterprise recycled Eternit’s used jute bags. The second one is South-West of Casale Monferrato, centered on the town of Cella Monte, where interviewed cases reported the widespread use of finely broken asbestos-cement waste materials for paving private roads, paths and court-yards. We have no similar explanation for the other clusters and indeed our analysis is intended to stimulate further investigations to identify and remediate possible hitherto unreported asbestos deposits.

As we know that models appeared to be sensible to distance threshold, selection of covariates and other modelling choices [39], different statistical methods were applied and compared. Changing the threshold of distance from the putative source and adjusting the models for different sets of covariates yielded consistent results, except for the raised incidence model.

Sensitivity analysis using a shorter lag time period (10 years) included similar number of subjects and produced similar results. This is consistent with the most commonly adopted estimate for the lag time in studies on MM [26]. It should however also be considered that the factory ceased its activity in 1986, that is 15 to 20 years before the diagnosis of our study cases, which may decrease any possible difference between using 20 or 10 years lag.

One limitation of our study is the higher level of non-response in controls: it affects the study power and, more importantly, may have led to selection bias. However, the difference between respondents and non-respondents was investigated recording socio-demographic and health-related data from administrative source and applying logistic regression models to investigate the variable associated with nonresponse. We observed that age and education were inversely associated with the response status, while gender was unrelated with it. Then, the association between asbestos exposure and MM was re-estimated and it remained unchanged, suggesting that no bias actually occurred [34]. Secondly, in the original analysis [22] asbestos exposure was considered as the sum of occupational, domestic and environmental component while in our study we adjusted only for occupational and domestic fibre concentration. Although, when we adjusted the models also for environmental component the distance estimates were similar and coherent.

## Conclusions

The study documents the large impact of an AC factory in Casale Monferrato, Italy on MM incidence in the area, with clustering of cases. It underlines the importance of assessing the impact of asbestos exposure not only among workers and family, but also among the community at large.

## Supplementary Information

**Additional file 1: Table S1.1.** Case control study on MM in Casale Monferrato area. Risk of MM of the pleura in relation to the distance of longest-held residence (after exclusion of 20 years before the date of diagnosis) from the AC plant. Absolute and relative frequencies of distance categories. Logistic models adjusted by age, sex, type of interview (*) and age, sex, type of interview and occupational and domestic asbestos exposure as continuous covariate (**), and age, sex, type of interview and domestic asbestos exposure as continuous covariate (***); odds ratios (OR), 95% confidence intervals (in brackets) and Akaike Information Criterion (AIC). **Table S1.2.** Case control study on MM in Casale Monferrato area. Risk of MM of the pleura in relation to the distance of longest-held residence (after exclusion of 20 years before the date of diagnosis) from the AC plant. Absolute and relative frequencies of distance categories. Logistic models adjusted by age, sex, type of interview (*); age, sex, type of interview and occupational and domestic asbestos exposure as continuous covariate (**) and age, sex, type of interview and domestic asbestos exposure as continuous covariate (***); odds ratios (OR), 95% confidence intervals (in brackets) and Akaike Information Criterion (AIC). **Table S1.3.** Case control study on MM in Casale Monferrato area. Risk of MM of the pleura in relation to the distance of longest-held residence (after exclusion of 20 years before the date of diagnosis) from the AC plant. Absolute and relative frequencies of distance categories and median [interquartile range] of distance. Logistic models adjusted by age, sex, type of interview, occupational exposure in a dichotomous way (*) and age, sex, type of interview and domestic exposure in a dichotomous way (**) and age, sex, type of interview and asbestos exposure (occupational, domestic and environmental) as continuous covariate (***) and age, sex, type of interview and asbestos exposure (domestic and environmental) as continuous covariate (****); odds ratios (OR), 95% confidence intervals (in brackets) and Akaike Information Criterion (AIC). **Figure S1.** Case control study on MM in Casale Monferrato area. Spatial distribution of the residences of cases (triangles) and controls (circles) in a geographic area of approximately 2500 km2 around Casale Monferrato (solid line). Residences are the longest-held among all residences of each individual after excluding 20 years before the date of diagnosis of the index case. The location of the AC plant (red triangle) and the center of the cluster found using the Kulldorf test (green triangle) in the town of Casale Monferrato are also indicated. [R Spatstat]. **Table S2.** Case control study on MM in Casale Monferrato area. Cuzick Edward test statistics (T_q_) and associate p-values based on 999 random labelling simulations for a variety of q values (3, 5, 7, 9, 11, 13, 15). **Table S3.1.** Case control study on MM in Casale Monferrato area. Risk of MM of the pleura in relation to the distance of longest-held residence (after exclusion of 10 years before the date of diagnosis) from the AC plant. Absolute and relative frequencies of distance categories and median [interquartile range] of distance. Logistic models adjusted by age, sex, type of interview (*) and age, sex, type of interview and occupational and domestic asbestos exposure as continuous covariate (**) or age, sex, type of interview and domestic asbestos exposure as continuous covariate (***); odds ratios (OR), 95% confidence intervals (in brackets) and Akaike Information Criterion (AIC). **Table S3.2.** Case control study on MM in Casale Monferrato area. Risk of MM of the pleura in relation to the distance of shorter residence (after exclusion of 20 years before the date of diagnosis) from the AC plant. Absolute and relative frequencies of distance categories and median [interquartile range] of distance. Logistic models adjusted by age, sex, type of interview (*) and age, sex, type of interview and occupational and domestic asbestos exposure as continuous covariate (**) or age, sex, type of interview and domestic asbestos exposure as continuous covariate (***); odds ratios (OR), 95% confidence intervals (in brackets) and Akaike Information Criterion (AIC).

## Data Availability

The dataset generated during and/or analyzed during the current study are not publicly available due to ethics reason.

## References

[1] IARC (International Agency for Cancer Research) (2012). A review of human carcinogens: arsenic, metals, fibres, and dusts. ARC Monogr Eval Carcinog Risks Hum C.

[2] Hodgson JT, Darnton A (2000). The quantitative risks of mesothelioma and lung cancer in relation to asbestos exposure. Ann Occup Hyg.

[3] Magnani C, Fubini B, Mirabelli D, Bertazzi PA, Bianchi C, Chellini E, Gennaro V, Marinaccio A, Menegozzo M, Merler E, Merletti F, Musti M, Pira E, Romanelli A, Terracini B, Zona A (2013). Pleural mesothelioma: epidemiological and public health issues. Report from the second italian consensus conference on pleural mesothelioma. Med Lav.

[4] Goswami E, Craven V, Dahlstrom DL, Alexander D, Mowat F (2013). Domestic asbestos exposure: a review of epidemiologic and exposure data. Int J Environ Res Public Health.

[5] Xu R, Barg FK, Emmett EA, Wiebe DJ, Hwang WT (2018). Association between mesothelioma and non-occupational asbestos exposure: systematic review and meta-analysis. Environ Health.

[6] Wagner JC, Sleggs CA, Marchand P (1960). Diffuse pleural mesothelioma and asbestos exposure in the North Western Cape Province. Br J Ind Med.

[7] Berry M (1997). Mesothelioma incidence and community asbestos exposure. Environ Res.

[8] Hansen J, De Klerk NH, Musk AW, Hobbs MS (1998). Environmental exposure to crocidolite and mesothelioma: exposure- response relationships. Am J Respir Crit Care Med.

[9] Howel D, Arblaster L, Swinburne L, Schweiger M, Renvoize E, Hatton P (1997). Routes of asbestos exposure and the development of mesothelioma in an English region. Occup Environ Med.

[10] Kielkowski D, Nelson G, Rees D (2000). Risk of mesothelioma from exposure to crocidolite asbestos: a 1995 update of a South African mortality study. Occup Environ Med.

[11] Kurumatani N, Kumagai S (2008). Mapping the risk of mesothelioma due to neighborhood asbestos exposure. Am J Respir Crit Care Med.

[12] Magnani C, Terracini B, Ivaldi C, Botta M, Mancini A, Andrion A (1995). Pleural malignant mesothelioma and non-occupational exposure to asbestos in Casale Monferrato. Italy. Occup Environ Med.

[13] Magnani C, Agudo A, González CA, Andrion A, Calleja A, Chellini E, Dalmasso P, Escolar A, Hernandez S, Ivaldi C, Mirabelli D, Ramirez J, Turuguet D, Usel M, Terracini B (2000). Multicentric study on malignant pleural mesothelioma and non-occupational exposure to asbestos. Br J Cancer.

[14] Maule MM, Magnani C, Dalmasso P, Mirabelli D, Merletti F, Biggeri A (2007). Modeling mesothelioma risk associated with environmental asbestos exposure. Environ Health Perspect.

[15] Musti M, Pollice A, Cavone D, Dragonieri S, Bilancia M (2009). The relationship between malignant mesothelioma and an asbestos cement plant environmental risk: a spatial case-control study in the city of Bari (Italy). Int Arch Occup Environ Health.

[16] Newhouse ML, Berry G (1976). Predictions of mortality from mesothelial tumours in asbestos factory workers. Br J Ind Med.

[17] Rees D, Myers JE, Goodman K, Fourie E, Blignaut C, Chapman R, Bachmann MO (1999). Case-control study of mesothelioma in South Africa. Am J Ind Med.

[18] Magnani C, Terracini B, Ivaldi C, Mancini A, Botta M (1996). Mortalita per tumori e altre cause tra i lavoratori del cemento-amianto a Casale Monferrato. Med Lav.

[19] CPO (Piedmont Reference Center for Epidemiology and Cancer Prevention). Malignant Mesothelioma Registry. https://www.cpo.it/workspace/files/2-incidenza-mesoteliomi-pleur-5ce2648477502.pdf. Accessed 13 Sept 2021.

[20] Magnani C, Ferrante D, Barone-Adesi F, Bertolotti M, Todesco A, Mirabelli D, Terracini B (2008). Cancer risk after cessation of asbestos exposure: a cohort study of Italian asbestos cement workers. Occup Environ Med.

[21] Ferrante D, Bertolotti M, Todesco A, Mirabelli D, Terracini B, Magnani C (2007). Cancer mortality and incidence of mesothelioma in a cohort of wives of asbestos workers in Casale Monferrato, Italy. Environ Health Perspect.

[22] Ferrante D, Mirabelli D, Tunesi S, Terracini B, Magnani C (2016). Pleural mesothelioma and occupational and non-occupational asbestos exposure: a case-control study with quantitative risk assessment. Occup Environ Med.

[23] Magnani C, Dalmasso P, Biggeri A, Ivaldi C, Mirabelli D, Terracini B (2001). Increased risk of malignant mesothelioma of the pleura after residential or domestic exposure to asbestos: a case-control study in Casale Monferrato, Italy. Environ Health Perspect.

[24] Arpa (2020). Mappatura amianto in Piemonte.

[25] Istat (2021). Demografia in cifre.

[26] Health Effects Institute (1991). Asbestos in public and commercial buildings: a literature review and synthesis of current knowledge.

[27] Pearce N (2016). Analysis of matched case-control studies. BMJ (Online).

[28] Kelsall JE, Diggle PJ (1995). Kernel estimation of relative risk. Bernoulli.

[29] Diggle PJ, Rowlingson BS (1994). A conditional approach to point process modelling of elevated risk. J R Statist Soc.

[30] Diggle PJ, Chetwynd AG (1991). Second-order analysis of spatial clustering for inhomogeneous populations. Biometrics.

[31] Kelsall JE, Diggle PJ (1998). Spatial variation in risk of disease: a nonparametric binary regression approach. J R Stat Soc Ser C Appl Stat.

[32] Kulldorff M (1997). A spatial scan statistic. Commun Stat Theory Methods.

[33] Cuzick J, Edwards R (1990). Spatial clustering for inhomogeneous populations. J R Stat Soc Series B (Methodological).

[34] Airoldi C, Ferrante D, Mirabelli D, Azzolina D, Magnani C (2020). Evaluation of nonresponse bias in a case–control study of pleural mesothelioma. Int J Environ Res Public Health.

[35] Kelsall JE, Diggle PJ (1995). Non-parametric estimation of spatial variation in relative risk. Stat Med.

[36] Mensi C, Riboldi L, De Matteis S, Bertazzi PA, Consonni D (2015). Impact of an asbestos cement factory on mesothelioma incidence: global assessment of effects of occupational, familial, and environmental exposure. Environ Int.

[37] Chiappino G, Sebastien P, Todaro A (1991). Atmospheric asbestos pollution in the urban environment: Milan, Casale Monferrato, Brescia, Ancona, Bologna and Florence. Med Lav.

[38] Marconi A, Cecchetti G, Barbieri M (1989). Airborne mineral fibre concentrations in an urban area near an asbestos-cement plant. IARC Sci Publ.

[39] Dreassi E, Lagazio C, Maule MM, Magnani C, Biggeri A (2008). Sensitivity analysis of the relationship between disease occurrence and distance from a putative source of pollution. Geospat Health.

