# Supplementary material for: Environmental asbestos exposure and clustering of malignant mesothelioma in community: a spatial analysis in a population-based case–control study
Source: Environ Health. 2021 Sep 15;20:103. doi: 10.1186/s12940-021-00790-3 (PMC8444609; doi:10.1186/s12940-021-00790-3)
Supplement: Supplementary file 1 — Additional file 1: Table S1.1. Case control study on MM in Casale Monferrato area. Risk of MM of the pleura in relation to the distance of longest-held residence (after exclusion of 20 years before the date of diagnosis) from the AC plant. Absolute and relative frequencies of distance categories. Logistic models adjusted by age, sex, type of interview (*) and age, sex, type of interview and occupational and domestic asbestos exposure as continuous covariate (**), and age, sex, type of interview and domestic asbestos exposure as continuous covariate (***); odds ratios (OR), 95% confidence intervals (in brackets) and Akaike Information Criterion (AIC). Table S1.2. Case control study on MM in Casale Monferrato area. Risk of MM of the pleura in relation to the distance of longest-held residence (after exclusion of 20 years before the date of diagnosis) from the AC plant. Absolute and relative frequencies of distance categories. Logistic models adjusted by age, sex, type of interview (*); age, sex, type of interview and occupational and domestic asbestos exposure as continuous covariate (**) and age, sex, type of interview and domestic asbestos exposure as continuous covariate (***); odds ratios (OR), 95% confidence intervals (in brackets) and Akaike Information Criterion (AIC). Table S1.3. Case control study on MM in Casale Monferrato area. Risk of MM of the pleura in relation to the distance of longest-held residence (after exclusion of 20 years before the date of diagnosis) from the AC plant. Absolute and relative frequencies of distance categories and median [interquartile range] of distance. Logistic models adjusted by age, sex, type of interview, occupational exposure in a dichotomous way (*) and age, sex, type of interview and domestic exposure in a dichotomous way (**) and age, sex, type of interview and asbestos exposure (occupational, domestic and environmental) as continuous covariate (***) and age, sex, type of interview and asbestos exposure (domestic and en [file 12940_2021_790_MOESM1_ESM.docx]

**Supplementary**

*Table S1.1. Case control study on MM in Casale Monferrato area. Risk of MM of the pleura in relation to the distance of longest-held residence (after exclusion of 20 years before the date of diagnosis) from the AC plant. Absolute and relative frequencies of distance categories. Logistic models adjusted by age, sex, type of interview (*) and age, sex, type of interview and occupational and domestic asbestos exposure as continuous covariate (**), and age, sex, type of interview and domestic asbestos exposure as continuous covariate (***); odds ratios (OR), 95% confidence intervals (in brackets) and Akaike Information Criterion (AIC).*

|  | |  |  |  |  |  |
| --- | --- | --- | --- | --- | --- | --- |
|  | | *All subjects* | | |  |  |
| **Distance from the AC plant (km)** | | **All**  **N=518** | **Cases**  **N=196** | **Controls**  **N=322** | **OR adjusted*** | **OR adjusted**** |
|  | |  |  |  |  |  |
| 0-3 | | 216 (41.70) | 115 (58.67) | 101 (31.37) | 11.46 (5.53-23.73) | 11.36 (5.48-23.55) |
| 3-5 | | 21 (4.05) | 11 (5.61) | 10 (3.11) | 7.05 (2.11-23.57) | 7.04 (2.11-23.56) |
| 5-7 | | 40 (7.72) | 20 (10.20) | 20 (6.21) | 10.09 (3.87-26.33) | 10.1 (3.87-26.35) |
| 7-9 | | 30 (5.79) | 17 (8.67) | 13 (4.04) | 12.22 (4.28-34.84) | 12.04 (4.21-34.4) |
| 9-11 | | 20 (3.86) | 7 (3.57) | 13 (4.04) | 3.25 (0.9-11.72) | 3.24 (0.9-11.72) |
| 11-13 | | 43 (8.30) | 10 (5.10) | 33 (10.25) | 2.44 (0.85-6.99) | 2.45 (0.85-7.01) |
| 13-15 | | 27 (5.21) | 4 (2.04) | 23 (7.14) | 1.7 (0.44-6.62) | 1.7 (0.44-6.61) |
| >15 | | 121 (23.36) | 12 (6.12) | 109 (33.85) | Ref | Ref |
|  | |  |  |  |  |  |
| **AIC** | |  |  |  | 520.93 | 522.69 |
|  | |  |  |  |  |  |
|  | *Non occupationally exposed subjects* | | | |  |  |
| **Distance from the AC plant (km)** | | **All**  **N=263** | **Cases**  **N=82** | **Controls**  **N=181** | **OR adjusted*** | **OR adjusted***** |
|  | |  |  |  |  |  |
| 0-3 | | 115 (43.73) | 53 (64.63) | 62 (34.25) | 14.39 (4.71-43.91) | 13.35 (4.35-40.93) |
| 3-5 | | 13 (4.94) | 6 (7.32) | 7 (3.87) | 9.91 (1.9-51.8) | 8.94 (1.73-46.29) |
| 5-7 | | 18 (6.84) | 6 (7.32) | 12 (6.63) | 5.23 (1.14-24.03) | 4.54 (0.95-21.66) |
| 7-9 | | 9 (3.42) | 5 (6.10) | 4 (2.21) | 23.23 (3.96-136.36) | 18.76 (3.05-115.58) |
| 9-11 | | 9 (3.42) | 3 (3.66) | 6 (3.31) | 3.86 (0.58-25.6) | 3.72 (0.56-24.71) |
| 11-13 | | 22 (8.37) | 4 (4.88) | 18 (9.94) | 3.93 (0.82-18.94) | 3.21 (0.65-15.95) |
| 13-15 | | 11 (4.18) |  | 11 (6.08) | - | - |
| >15 | | 66 (25.10) | 5 (6.10) | 61 (33.70) | Ref | Ref |
|  | |  |  |  |  |  |
|  | |  |  |  | 252.52 | 251.28 |

*Table S1.2. Case control study on MM in Casale Monferrato area. Risk of MM of the pleura in relation to the distance of longest-held residence (after exclusion of 20 years before the date of diagnosis) from the AC plant. Absolute and relative frequencies of distance categories. Logistic models adjusted by age, sex, type of interview (*); age, sex, type of interview and occupational and domestic asbestos exposure as continuous covariate (**) and age, sex, type of interview and domestic asbestos exposure as continuous covariate (***); odds ratios (OR), 95% confidence intervals (in brackets) and Akaike Information Criterion (AIC).*

|  | |  |  |  | |  |  |
| --- | --- | --- | --- | --- | --- | --- | --- |
|  | | *All subjects* | | | |  |  |
| **Distance from the AC plant (meters)** | | **All**  **N=518** | **Cases**  **N=196** | **Controls**  **N=322** | | **OR adjusted*** | **OR adjusted**** |
|  | |  |  |  | |  |  |
| 0-500 | | 8 (1.54) | 8 (4.08) | 0 (0.00) | | - | - |
| 500-1000 | | 18 (3.47) | 14 (7.14) | 4 (1.24) | | 13.58 (4.22-43.73) | 13.7 (4.25-44.11) |
| 1000-1500 | | 84 (16.22) | 38 (19.39) | 46 (14.29) | | 2.08 (1.19-3.63) | 1.99 (1.14-3.5) |
| 1500-2000 | | 57 (11.00) | 31 (15.82) | 26 (8.07) | | 2.94 (1.55-5.57) | 2.92 (1.54-5.54) |
| >2000 | | 351 (67.76) | 105 (53.57) | 246 (76.40) | | Ref | Ref |
|  | |  |  |  | |  |  |
| **AIC** | |  |  |  | | 545.30 | 546.19 |
|  | |  |  |  | |  |  |
|  | |  |  |  | |  |  |
|  | *Non occupationally exposed subjects* | | | |  |  |  |
| **Distance from the AC plant (km)** | | **All**  **N=263** | **Cases**  **N=82** | **Controls**  **N=181** | | **OR adjusted*** | **OR adjusted***** |
|  | |  |  |  | |  |  |
| 0-500 | | 3 (1.14) | 3 (3.66) | 0 (0.00) | |  |  |
| 500-1000 | | 13 (4.94) | 10 (12.20) | 3 (1.66) | | 17.82 (4.38-72.44) | 18.45 (4.5-75.62) |
| 1000-1500 | | 44 (16.73) | 14 (17.07) | 30 (16.57) | | 1.15 (0.49-2.7) | 1.25 (0.53-2.95) |
| 1500-2000 | | 31 (11.79) | 12 (14.63) | 19 (10.50) | | 2.25 (0.89-5.7) | 2.24 (0.86-5.78) |
| >2000 | | 172 (65.40) | 43 (52.44) | 129 (71.27) | | Ref | Ref |
|  | |  |  |  | |  |  |
| **AIC** | |  |  |  | | 258.11 | 255.07 |

*Table S1.3 Case control study on MM in Casale Monferrato area. Risk of MM of the pleura in relation to the distance of longest-held residence (after exclusion of 20 years before the date of diagnosis) from the AC plant. Absolute and relative frequencies of distance categories and median [interquartile range] of distance. Logistic models adjusted by age, sex, type of interview, occupational exposure in a dichotomous way (*) and age, sex, type of interview and domestic exposure in a dichotomous way (**) and age, sex, type of interview and asbestos exposure (occupational, domestic and environmental) as continuous covariate (***) and age, sex, type of interview and asbestos exposure (domestic and environmental) as continuous covariate (****); odds ratios (OR), 95% confidence intervals (in brackets) and Akaike Information Criterion (AIC).*

|  | |  |  | |  |
| --- | --- | --- | --- | --- | --- |
|  | | *All subjects* | | |  |
| **Distance from the AC plant (km)** | | **OR adjusted*** | **OR adjusted**** | | **OR adjusted***** |
|  | |  |  | |  |
| 0-5 | | 11.75 (5.67-24.37) | 10.11 (4.9-20.85) | | 10.8 (5.26-22.19) |
| 5-10 | | 10.39 (4.47-24.16) | 9.54 (4.08-22.32) | | 10.41 (4.51-24.06) |
| 10-15 | | 2.22 (0.91-5.41) | 2.22 (0.91-5.38) | | 2.2 (0.9-5.34) |
| >15 | | Ref | Ref | | Ref |
| **AIC** | | 504.68 | 505.98 | | 515.39 |
|  | |  |  | |  |
| Distance, Km | | 0.87 (0.84-0.9) | 0.88 (0.85-0.91) | | 0.88 (0.84-0.91) |
| **AIC** | | 514.39 | 505.53 | | 516.58 |
|  | |  |  | |  |
|  | |  |  | |  |
|  | *Non-occupationally exposed subjects* | | |  |  |
| **Distance from the AC plant (km)** | |  | **OR adjusted**** | | **OR adjusted****** |
|  | |  |  | |  |
| 0-5 | |  | 11.66 (3.74-36.37) | | 7.39 (2.34-23.39) |
| 5-10 | |  | 7.57 (1.92-29.85) | | 5.72 (1.46-22.38) |
| 10-15 | |  | 2.74 (0.67-11.28) | | 2.31 (0.56-9.49) |
| >15 | |  | Ref | | Ref |
| **AIC** | |  | 242.79 | | 237.55 |
|  | |  |  | |  |
| Distance, Km | |  | 0.87 (0.82-0.92) | | 0.89 (0.84-0.95) |
| **AIC** | |  | 237.13 | | 233.77 |

*Figure S1. Case control study on MM in Casale Monferrato area. Spatial distribution of the residences of cases (triangles) and controls (circles) in a geographic area of approximately 2500 km2 around Casale Monferrato (solid line). Residences are the longest-held among all residences of each individual after excluding 20 years before the date of diagnosis of the index case. The location of the AC plant (red triangle) and the center of the cluster found using the Kulldorf test (green triangle) in the town of Casale Monferrato are also indicated. [R Spatstat]*


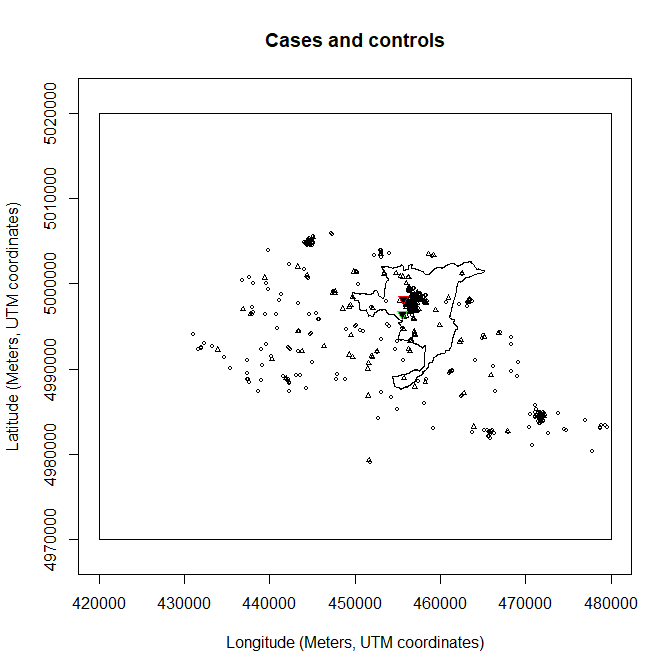


*Table S2. Case control study on MM in Casale Monferrato area. Cuzick Edward test statistics (T_q_) and associate p-values based on 999 random labelling simulations for a variety of q values (3, 5, 7, 9, 11, 13, 15).*

|  |  |  |
| --- | --- | --- |
|  |  |  |
| **Number of nearest neighbors (q)** | **Tq** | **p-value** |
| 3 | 312 | <0.001 |
| 5 | 511 | <0.001 |
| 7 | 697 | <0.001 |
| 9 | 884 | <0.001 |
| 11 | 1073 | <0.001 |
| 13 | 1255 | <0.001 |
| 15 | 1414 | <0.001 |
|  |  |  |
|  |  |  |
|  |  |  |
| **Contrast** | **Value** | **p-value** |
| T_5_-T_3_ | 199 | <0.001 |
| T_7_-T_3_ | 385 | <0.001 |
| T_9_-T_3_ | 572 | <0.001 |
| T_11_-T_3_ | 761 | <0.001 |
| T_13_-T_3_ | 943 | <0.001 |
| T_15_-T_3_ | 1102 | <0.001 |
| T_7_-T_5_ | 186 | 0.004 |
| T_9_-T_5_ | 373 | <0.001 |
| T_11_-T_5_ | 562 | <0.001 |
| T_13_-T_5_ | 744 | <0.001 |
| T_15_-T_5_ | 903 | <0.001 |
| T_9_-T_7_ | 187 | <0.001 |
| T_11_-T_7_ | 376 | <0.001 |
| T_13_-T_7_ | 558 | <0.001 |
| T_15_-T_7_ | 717 | <0.001 |
| T_11_-T_9_ | 189 | <0.001 |
| T_13_-T_9_ | 371 | <0.001 |
| T_15_-T_9_ | 530 | <0.001 |
| T_13_-T_11_ | 182 | 0.005 |
| T_15_-T_11_ | 341 | 0.008 |
| T_13_-T_15_ | 159 | 0.210 |

*Table S3.1 Case control study on MM in Casale Monferrato area. Risk of MM of the pleura in relation to the distance of longest-held residence (after exclusion of 10 years before the date of diagnosis) from the AC plant. Absolute and relative frequencies of distance categories and median [interquartile range] of distance. Logistic models adjusted by age, sex, type of interview (*) and age, sex, type of interview and occupational and domestic asbestos exposure as continuous covariate (**) or age, sex, type of interview and domestic asbestos exposure as continuous covariate (***); odds ratios (OR), 95% confidence intervals (in brackets) and Akaike Information Criterion (AIC).*

|  | |  |  |  |  |  |
| --- | --- | --- | --- | --- | --- | --- |
|  | | *All subjects* | | |  |  |
| **Distance from the AC plant (km)** | | **All**  **N=529** | **Cases**  **N=198** | **Controls**  **N=331** | **OR adjusted*** | **OR adjusted**** |
| 0-5 | | 237 (44.80) | 124 (62.63) | 113 (34.14) | 10.26 (5.09-20.67) | 10.17 (5.04-20.5) |
| 5-10 | | 79 (14.93) | 42 (21.21) | 37 (11.18) | 10.72 (4.76-24.12) | 10.65 (4.73-23.99) |
| 10-15 | | 86 (16.26) | 19 (9.60) | 67 (20.24) | 2.34 (0.99-5.54) | 2.34 (0.99-5.54) |
| >15 | | 127 (24.01) | 13 (6.57) | 114 (34.44) | Ref | Ref |
| **AIC** | |  |  |  | 523.76 | 525.45 |
|  | |  |  |  |  |  |
| Distance, Km | | 6.18 [12.63] | 2.13 [6.15] | 11.38 [16.09] | 0.88 (0.85-0.91) | 0.88 (0.85-0.91) |
| **AIC** | |  |  |  | 526.03 | 527.71 |
|  | |  |  |  |  |  |
|  | |  |  |  |  |  |
|  | *Non-occupationally exposed subjects* | | | |  |  |
| **Distance from the AC plant (km)** | | **All**  **N=270** | **Cases**  **N=83** | **Controls**  **N=187** | **OR adjusted*** | **OR adjusted***** |
|  | |  |  |  |  |  |
| 0-5 | | 127 (47.04) | 57 (68.67) | 70 (37.43) | 13.01 (4.36-38.87) | 12 (4-36.03) |
| 5-10 | | 34 (12.59) | 14 (16.87) | 20 (10.70) | 10 (2.81-35.58) | 8.66 (2.38-31.51) |
| 10-15 | | 41 (15.19) | 7 (8.43) | 34 (18.18) | 3.38 (0.88-12.93) | 2.97 (0.77-11.52) |
| >15 | | 68 (25.19) | 5 (6.02) | 63 (33.69) | Ref | Ref |
| **AIC** | |  |  |  | 259.67 | 257.85 |
|  | |  |  |  |  |  |
| Distance, Km | | 5.89 [13.56] | 2.07 [4.91] | 10.36 [15.98] | 0.87 (0.82-0.92) | 0.87 (0.82-0.92) |
| **AIC** | |  |  |  | 255.85 | 253.37 |

*Table S3.2 Case control study on MM in Casale Monferrato area. Risk of MM of the pleura in relation to the distance of shorter residence (after exclusion of 20 years before the date of diagnosis) from the AC plant. Absolute and relative frequencies of distance categories and median [interquartile range] of distance. Logistic models adjusted by age, sex, type of interview (*) and age, sex, type of interview and occupational and domestic asbestos exposure as continuous covariate (**) or age, sex, type of interview and domestic asbestos exposure as continuous covariate (***); odds ratios (OR), 95% confidence intervals (in brackets) and Akaike Information Criterion (AIC).*

|  | | *All subjects* | | |  |  |
| --- | --- | --- | --- | --- | --- | --- |
| **Distance from the AC plant (km)** | | **All**  **N=518** | **Cases**  **N=196** | **Controls**  **N=322** | **OR adjusted*** | **OR adjusted**** |
|  | |  |  |  |  |  |
| 0-5 | | 282 (54.44) | 144 (73.47) | 138 (42.86) | 11.59 (5.36-25.08) | 11.48 (5.3-24.86) |
| 5-10 | | 56 (10.81) | 29 (14.80) | 27 (8.39) | 9.04 (3.52-23.22) | 9.06 (3.53-23.27) |
| 10-15 | | 73 (14.09) | 13 (6.63) | 60 (18.63) | 2.03 (0.76-5.47) | 2.04 (0.76-5.48) |
| >15 | | 107 (20.66) | 10 (5.10) | 97 (30.12) | Ref | Ref |
| **AIC** | |  |  |  | 517.33 | 519.00 |
|  | |  |  |  |  |  |
| Distance, Km | | 2.70 [11.70] | 1.38 [4.35] | 8.12 [15.00] | 0.87 (0.83-0.9) | 0.87 (0.83-0.9) |
| **AIC** | |  |  |  | 512.52 | 514.27 |
|  | |  |  |  |  |  |
|  | *Non-occupationally exposed subjects* | | | |  |  |
| **Distance from the AC plant (km)** | | **All**  **N=263** | **Cases**  **N=82** | **Controls**  **N=181** | **OR adjusted*** | **OR adjusted***** |
|  | |  |  |  |  |  |
| 0-5 | | 148 (56.27) | 63 (76.83) | 85 (46.96) | 14.04 (4.21-46.88) | 12.87 (3.83-43.2) |
| 5-10 | | 22 (8.37) | 9 (10.98) | 13 (7.18) | 8.55 (1.9-38.34) | 7.1 (1.53-33.05) |
| 10-15 | | 37 (14.07) | 6 (7.32) | 31 (17.13) | 3.4 (0.77-15.07) | 2.98 (0.66-13.41) |
| >15 | | 56 (21.29) | 4 (4.88) | 52 (28.73) | Ref | Ref |
| **AIC** | |  |  |  | 256.53 | 254.45 |
|  | |  |  |  |  |  |
| Distance, Km | | 2.53 [11.70] | 1.37 [3.10] | 6.03 [14.77] | 0.86 (0.81-0.92) | 0.86 (0.81-0.92) |
| **AIC** | |  |  |  | 251.69 | 249.11 |
